# Wet Ageing of Chilled Young-Giraffe (*Giraffa camelopardalis angolensis*) Meat as Influenced by Sex and Muscle †

**DOI:** 10.3390/foods15071236

**Published:** 2026-04-04

**Authors:** Bianca L. Silberbauer, Tersia Kokošková, Daniel Bureš, Radim Kotrba, Philip E. Strydom, Martin Kidd, Louwrens C. Hoffman

**Affiliations:** 1 Department of Animal Sciences, University of Stellenbosch, Private Bag X1, Matieland, Stellenbosch 7602, South Africa; biancasilberbauer95@gmail.com (B.L.S.); kokoskova@ftz.czu.cz (T.K.); pestrydom@sun.ac.za (P.E.S.); 2 Department of Animal Science and Food Processing, Faculty of Tropical AgriSciences, Czech University of Life Sciences Prague, Kamýcká 129, 16500 Prague, Czech Republic; kotrba@ftz.czu.cz; 3 Department of Food Science, Faculty of Agrobiology, Food and Natural Sciences, Czech University of Life Sciences Prague, Kamýcká 129, 16500 Prague, Czech Republic; bures.daniel@vuzv.cz; 4 Department of Ethology, Institute of Animal Science, Přátelství 815, Praha Uhříněves, 10400 Prague, Czech Republic; 5 Centre for Statistical Consultation, Department of Statistics and Actuarial Sciences, University of Stellenbosch, Private Bag X1, Matieland, Stellenbosch 7602, South Africa; mkidd@sun.ac.za; 6 Centre for Nutrition and Food Sciences, Queensland Alliance for Agriculture and Food Innovation (QAAFI), The University of Queensland, Digital Agricultural Building, 8115. Office 110, Gatton 4343, Australia

**Keywords:** post mortem ageing, game meat, giraffe, tenderness, vacuum ageing, meat colour, Warner–Bratzler shear force

## Abstract

This exploratory study determined the ideal ageing period for optimum tenderness of *Longissimus thoracis et lumborum* (LTL), *Semimembranosus* (SM) and *Biceps femoris* (BF) steaks from male and female giraffe. The muscles of eight male and seven female giraffes were divided into 10 steaks each, and each steak was randomly allocated to age for 1, 5, 9, 14, 18, 22, 26, 30, 34 or 38 days in vacuum-sealed bags at ±4 °C. At each time point, the pH, surface colour, purge loss, cooking loss and Warner–Bratzler shear force (WBSF) were determined for the respective steaks. Significant interactions between the sex, muscle and days post mortem were observed for the Warner–Bratzler shear force (WBSF), CIE a*, CIE b*, hue-angle, and chroma, while the CIE L* values were affected by an interaction between muscle type and days post mortem. The pH showed three distinct phases: over Days 1–9, the pH (~5.54–5.55) was stable; over Days 14–22, it declined; and the pH dropped more sharply between Days 22 and 26 (~5.42 to ~5.32), before plateauing. The purge loss initially increased rapidly, after which the rate decreased during the ageing period; however, the cooking loss, which was affected only by muscle, remained constant throughout. The tenderness improved until Day 22 across all three muscle types (19.1 ± 0.30 N), after which it plateaued. The colour improved, in terms of redness and saturation, until Day 18 (L* = 44.1 ± 0.29; a* = 15.7 ± 0.19; b* = 15.3 ± 0.08; hue-angle = 44.8 ± 0.39; chroma = 22.0 ± 0.15); thereafter, discolouration occurred. Vacuum-ageing giraffe meat for 14–22 days is recommended to improve tenderness and colour and minimise the negative effects of increased purge loss. This recommendation is based on instrumental measurements with discolouration as a major determinant of acceptability. It is suggested that future research validate this through sensory evaluation and microbial analyses.

## 1. Introduction

There is a growing demand for food production to feed the ever-increasing population of southern Africa, with the population of Africa predicted to surpass two billion within the next few decades, which will leave southern Africa in a dire state, as it is already a net importer of food, with a poor economy and widespread undernourishment [1]. As animal products contain many nutrients in more readily available forms than plant sources [2,3,4,5,6], it is vital to optimise their production. It is also essential to utilise available land wisely for animal production, as Africa is mainly dry and arid, conditions to which many domestic livestock species are not adapted. However, Africa has abundant indigenous species that are naturally adapted to its climatic conditions and available vegetation. These species can help address food insecurity [7,8,9,10,11,12]. Although there is limited research on the meat quality of these various species, wet ageing has been shown to increase the tenderness of eland (*Taurotragus oryx*) and African buffalo (*Syncerus caffer*) meat—two large ungulate species [1,13,14,15]

Consumer preferences and perceptions pose a challenge to increasing the production of indigenous species for meat [16,17,18,19,20]. Consumers have become accustomed to meat from domestic species and may discriminate against meat that does not look or taste the same. Game meat tends to be darker than traditional commercially produced meat [21,22], which may deter consumers from purchasing it. There is also concern among many consumers about the safety of game meat [17,23].

Due to the variation in the methods used to slaughter game species, there is often a large amount of variation in the quality of game meat, as the quality is strongly affected by the ante mortem stress [24], which depends on the efficiency of the hunters/culling team. Consumers, therefore, often perceive game meat as tough and dry due to product variation, the lack of quality standards, and limited knowledge of how to prepare it [25,26]. To increase the marketability of game meat, it is necessary to consistently produce high-quality meat and counter consumer perceptions of its toughness. Post mortem ageing of meat is known to improve its tenderness through a combination of enzymatic activities that break down structural proteins over time [27,28,29,30]. The ageing period to optimal tenderness is species-dependent, as enzyme concentrations vary by species [31,32,33]. The optimum ageing period for each species needs to be investigated to improve the quality of game meat.

While giraffe numbers are dropping across central and east Africa, in southern Africa, specifically in South Africa and Namibia, their populations are growing exponentially due to an increasing number of game ranches breeding them [34,35]. As predators in the wild keep their populations in check, when they are fenced into camps with no natural predators, their populations grow exponentially, necessitating culling to prevent exceeding the carrying capacity [36]. Due to their large bulk, each animal yields a large amount of meat [37,38]; however, more research is needed into the quality of giraffe meat. According to accounts from farmers who have eaten fresh giraffe meat, it is tough and is usually used only in processed products, such as boerewors (a traditional South African sausage). However, the tenderness of the fresh giraffe meat was found to be comparable with that of the fresh meat of other game species [39] and was classified as tender by conventional physical standards, as it had a shear value < 43 N [40]. However, a large amount of variation was observed among the samples, with some classified as intermediate tender (>43 N) [39]. Therefore, post mortem ageing may produce a more uniform product of acceptable tenderness. This exploratory study aims to determine the ageing time to optimal tenderness for giraffe meat to improve its eating quality, while maintaining sufficient moisture for juiciness and an appealing colour, to produce a product acceptable to consumers. In previous research, the African buffalo showed an optimal wet ageing period of 25 days [15]; it was decided to evaluate the influence of wet ageing on giraffe meat over a 28-day period. Due to the challenges of conducting research on large, free-roaming wild ungulates in non-laboratory environments, this exploratory investigation and conclusion are based on instrumental analyses rather than consumer evaluations.

## 2. Materials and Methods

### 2.1. Experimental Location and Animals

Fifteen young giraffes (8 male, 7 female; age ≈ 3½ years old as estimated by an experienced wildlife manager) were harvested on Mount Etjo farm in the Otjozondjupa region of Namibia as part of their annual cull to control the population growth. The giraffes were culled by headshot and exsanguinated in the field (Ethical approval: ACU-2018-7366, Stellenbosch University; Namibian Shoot and sell permit number: 118690) before being transported back to the abattoir, where they were skinned, eviscerated and dressed as described by Ledger [41]. Refer to Silberbauer et al. [38] for more details on the dressing and yields of the giraffe.

### 2.2. Processing and Sampling

The carcasses were chilled for approximately 24 h at ±4 °C before the muscles were removed and processed further. Three muscles from the right side of each carcass were removed for post mortem ageing: the *Longissimus thoracis et lumborum* muscle (LTL), *Semimembranosus* muscle (SM) and *Biceps femoris* muscle (BF). Ten steaks of approximately 100–200 g, approximately 2 cm thick, were cut from each animal’s muscles. Each steak was randomly allocated to an ageing day from 1 to 38. It is acknowledged that the steak-to-steak variation within a muscle will contribute to residual error. The Day 1 steaks were processed immediately, as described in Hoffman et al. [39]. The other steaks were vacuum sealed in composite plastic bags (70 μm nylon and polyethylene; oxygen permeability of 30 cm^3^/m^2^/24 h/1 atm, carbon dioxide permeability of 105 cm^3^/m^2^/24 h/1 atm and moisture vapour transfer rate of 2.2 g/m^2^/24 h/1 atm) and kept refrigerated at ±4 °C until the respective ageing day.

It was decided to give the giraffe a long 38-day ageing period, as farmers who only use the meat in processed products had described it as tough (Table 1).

### 2.3. Physical Analysis

The cumulative purge loss and cooking loss percentages determined the total moisture loss. The purge loss was measured by blotting the steak from each muscle for the respective day dry with paper towelling and weighing it. The purge loss percentage was calculated as the loss of each steak’s initial weight.

The pH of each steak on the respective ageing day was measured after determining the purge loss by the methods described in Hoffman et al. [39], using a (two-point calibration using standard buffers of pH 4 and pH 7) Accsen pH 70+ DHS^®^ portable pH meter (Accsen Instrumental, Barcelona, Spain).

The colour of each steak was measured after each steak had been weighed back for purge loss and allowed to bloom for ±30 min. A calibrated Colour-guide 45°/0° colorimeter (model 6801, BYK-Gardner GmbH, Geretsried, Germany; aperture diameter size: 11 mm; illuminant/observer angle: D-65/10°) was used, taking five measurements at random on the cut surface as described in Hoffman et al. [39]. The reported lightness (CIE L*), red–green spectrum (CIE a*) and blue–yellow spectrum (CIE b*) values were then used to calculate the hue-angle (colour definition) and chroma values (saturation/colour intensity) for each steak by using the following equations:

$$Hue-angle\;\left({}^{\circ}\right)={tan}^{-1}\left(\frac{b^{*}}{a^{*}}\right)$$

$$Chroma\;\left(C^{*}\right)=\sqrt{\left(a^{*2}+b^{*2}\right)}$$

The cooking loss for each steak was determined in the same manner as described in Hoffman et al. [39] by cooking each animal’s three steaks together (each muscle individually placed inside a bag) in a preheated water bath at 80 °C for 1 h; after the purge loss, pH and colour were measured. It is acknowledged that these temperatures and durations could be severe and may contribute to higher cooking losses; however, this protocol has been followed for meat from different ungulate species and will allow comparison of results across species.

After the cooking loss had been determined, the cooked steaks were used to determine the Warner–Bratzler shear force (WBSF) as an indication of the tenderness of each muscle on the respective day. A portable Instron machine fitted with a Warner–Bratzler blade was used, and the WBSF of six 1.27 cm diameter cylindrical cores per steak was determined [39]. The recorded measurements, in kg/1.27 cm ϕ, were then converted to Newtons (N) using the following equation:

$$Warner-Bratzler\;shear\;force\;\left(N\right)=\frac{\left(F\;\times \;9.81\right)}{Area}$$

where F=kg/1.27 cm ϕ

$$and\;area=\pi {\left(\frac{1.27}{2}\right)}^{2}$$

### 2.4. Statistical Analysis

Statistica Version 14.2 (2023) and the R lme4 package were used to perform statistical analyses on the data. A mixed-model ANOVA was used with animals, animal × muscle, and animal × day as random effects, and age, muscle, sex, and day as fixed effects. For post hoc testing, Fisher’s Least Significant Difference (LSD) was used for multiple-comparison testing [42]. Outliers were identified using normal probability plots and were detected in only one instance. In this case, winsorizing was performed to reduce the potential impact of outliers. The winsorized result, however, did not deviate substantially from the original. The 5% probability level (*p* ≤ 0.05) was used to indicate significance. It should be noted that in such a large experimental design, correcting for multiple testing becomes problematic, as more conservative methods tend to overcompensate for type I errors, thereby increasing the risk of type II errors. This study should be viewed as exploratory, and no strong conclusions are made (considering the possibility of type I errors). 

## 3. Results

The age of the giraffe did not affect any of the parameters (Table 2) and will not be discussed further. For the WBSF, CIE a*, CIE b*, hue-angle, and chroma, there were significant interactions between sex, muscle, and day (Table 2). Due to the minimal impact of sex on these parameters, only the second-order interaction (Muscle × Day) will be discussed further (see Supplementary Data). A possible reason for the low influence of sex on the parameters could be attributed to the giraffe reaching a physiological puberty age, and therefore, secondary sexual characteristics and impacts are only then beginning to manifest themselves. Discussion with the producer highlighted that the target giraffes are around 3–4 years old for meat use in their restaurant, as mince, burgers, sausages, or steaks. CIE L* was also affected by a second-order interaction of muscle and day. pH and purge loss were affected only by day, and cooking loss was affected only by muscle. Cognisance should be taken of the large standard error of the means in the results. This phenomenon was most probably caused by the inherent variability in these quality parameters within a muscle.

**Figure 1:**
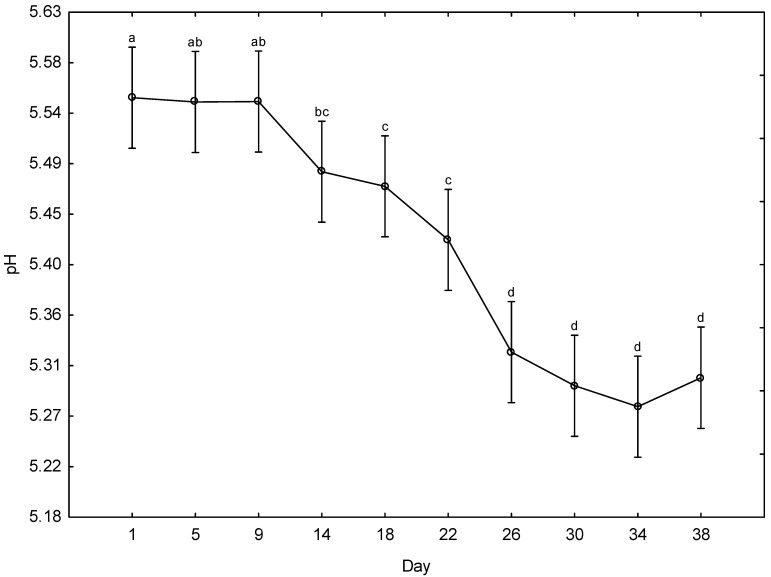
The effect of days post mortem on the pH of giraffe meat (all animals, all muscles, days) during a 38-day ageing period. ^a–d^ Means (±s.e.) with different superscripts differ from one another (*p* ≤ 0.05).

The days post mortem was the only factor affecting the pH of the giraffe meat. Figure 1 indicates the pH of giraffe meat (pooled across all animals and muscles) over 38 days post mortem. There is a gradual, stepwise decline from ~5.55 to ~5.28, with three distinct phases. Phase 1—Days 1–9 (pH ~5.54–5.55, group “^a/ab^”): pH is stable and does not differ significantly. The shift from “^ab^” to “^bc^” between Days 9 and 14 marks the first statistically significant break. Phase 2—A significant decline begins between Days 14 and 22 (pH ~5.49–5.42, group “^bc/c^”). Phase 3—Days 26–38 (pH ~5.27–5.32, group “^d^”): pH drops more significantly between Days 22 and 26 (~5.42 to ~5.32) and then plateaus. The large error bars (±0.05–0.10) indicate substantial variability across muscles and individual animals. This is expected given the pooled dataset.

**Figure 2:**
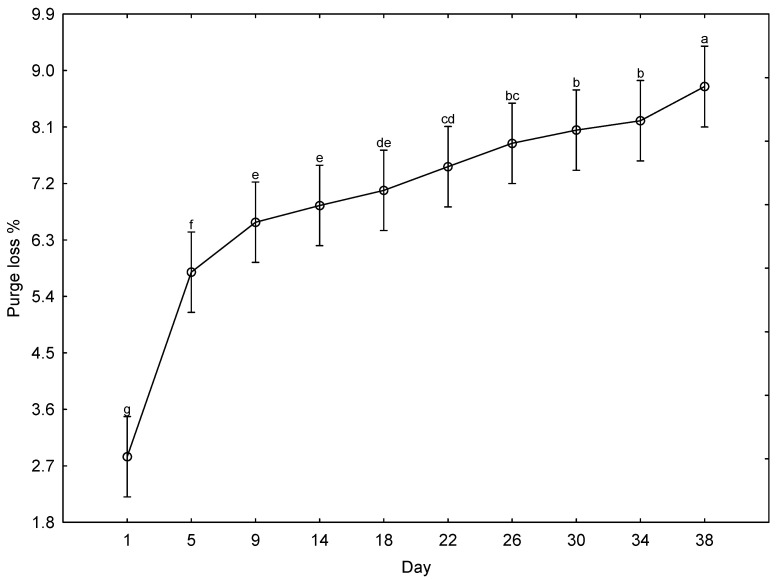
The effect of days post mortem on the purge loss % of giraffe meat (all animals, all muscles, days) during a 38-day ageing period. ^a–g^ Means (±s.e.) with different superscripts differ from one another (*p* ≤ 0.05).

Days post mortem was the only factor affecting the cumulative purge loss of the giraffe meat (Figure 2). Day 1 had the lowest purge loss (2.8 ± 0.31%), and Day 5 had more than double this loss (5.8 ± 0.31%). The purge loss increased steadily from Day 5 throughout the ageing period, reaching 8.7 ± 0.31% on Day 38.

**Figure 3:**
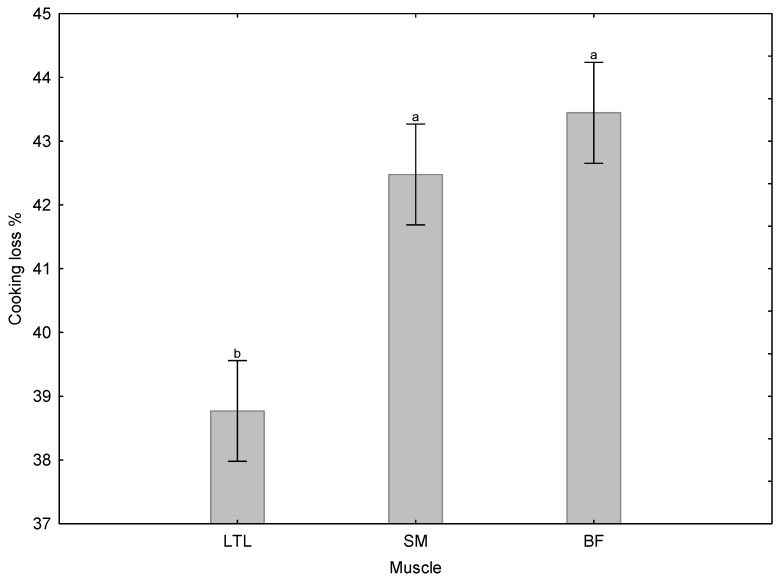
The effect of muscle type on the cooking loss % of giraffe meat during a 38-day ageing period. ^a,b^ Means (±s.e.) with different superscripts differ from one another (*p* ≤ 0.05).

The cooking loss was not affected by days post mortem; it was only affected by the muscle type (Figure 3). The LTL had the lowest cooking loss (38.8 ± 0.39%), and the SM (42.5 ± 0.39%) and BF (43.4 ± 0.39%) both had significantly higher cooking losses throughout the ageing period.

The WBSF of the meat was affected by a third-order interaction between the sex, muscle and day (Table 2). Despite this interaction, there were few significant differences in sex or muscle at any time point during the ageing period. Sex did not have an overall effect on the WBSF (*p* = 0.177), but muscle did (*p* = 0.007). The interaction between muscle and days post mortem is therefore shown in Table 3. Overall, WBSF decreased sharply during the first five days of the ageing period and continued to decline until Day 22, after which it plateaued for the remainder of the ageing period (Table 3). The meat from the SM (22.1 ± 0.65 N) had a higher WBSF than from the LTL (20.3 ± 0.65 N) or the BF (20.5 ± 0.65 N) for the duration of the ageing period.

**Figure 4:**
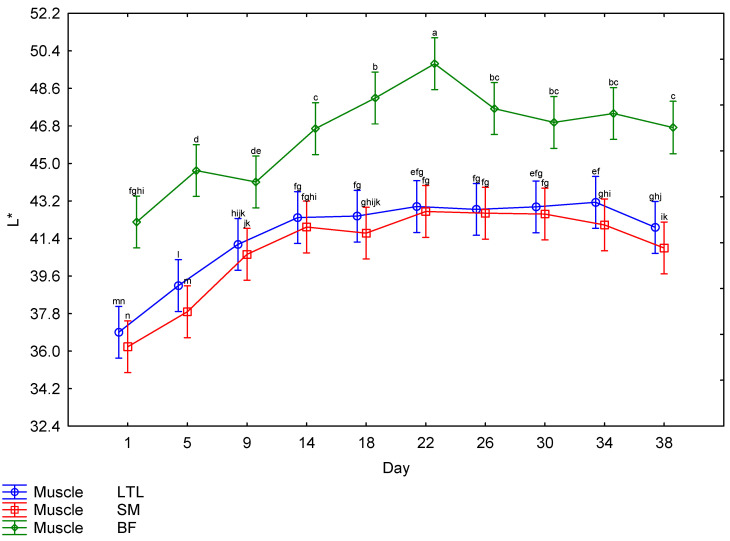
The effect of the interaction between muscle and day on the L* values of giraffe meat during a 38-day ageing period. ^a–n^ Means (±s.e.) with different superscripts differ from one (*p* ≤ 0.05).

**Figure 5:**
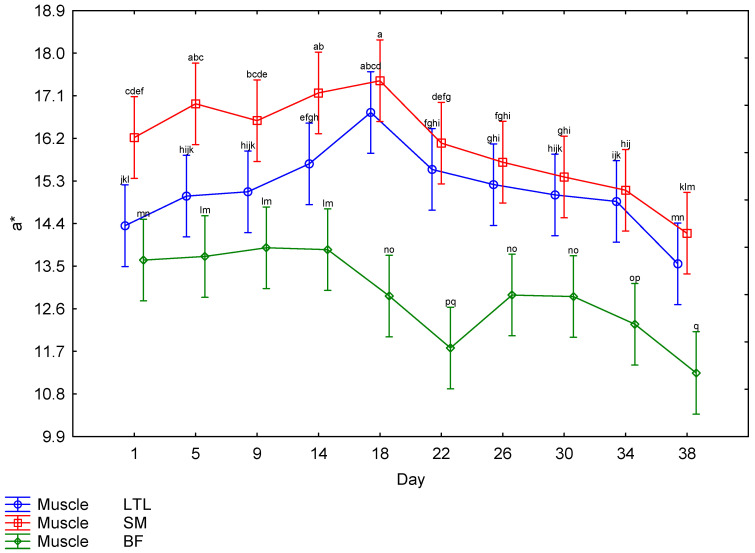
The effect of the interaction between muscle and day on the a* values of giraffe meat during a 38-day ageing period. ^a–q^ Means (±s.e.) with different superscripts differ from one another (*p* ≤ 0.05).

**Figure 6:**
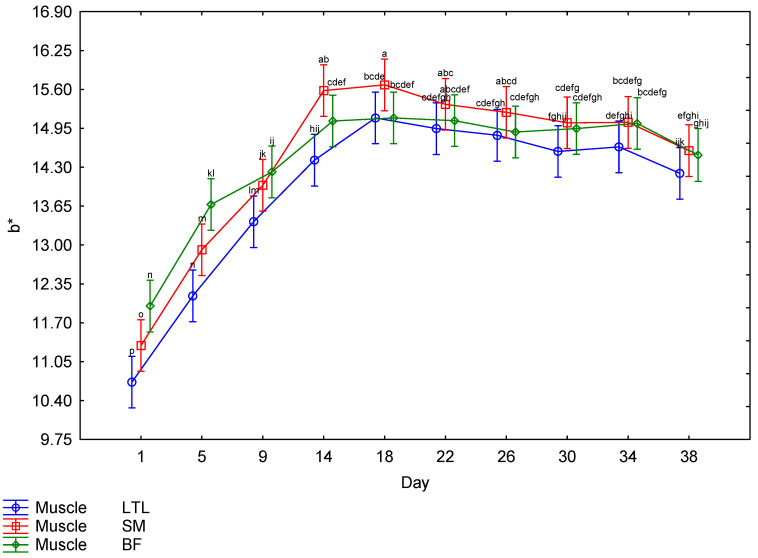
The effect of the interaction between muscle and day on the b* values of giraffe meat during a 38-day ageing period. ^a–p^ Means (±s.e.) with different superscripts differ from one another (*p* ≤ 0.05).

**Figure 7:**
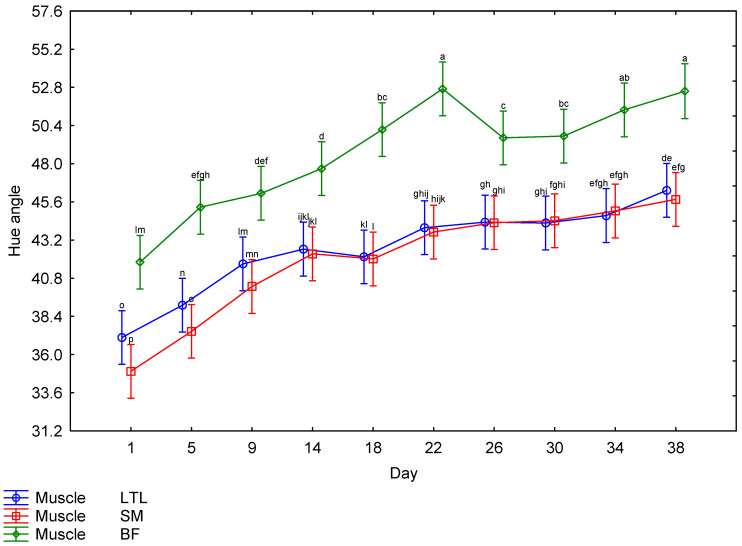
The effect of the interaction between muscle and days post mortem on the hue-angle of giraffe meat during a 38-day ageing period. ^a–p^ Means (±s.e.) with different superscripts differ from one another (*p* ≤ 0.05).

**Figure 8:**
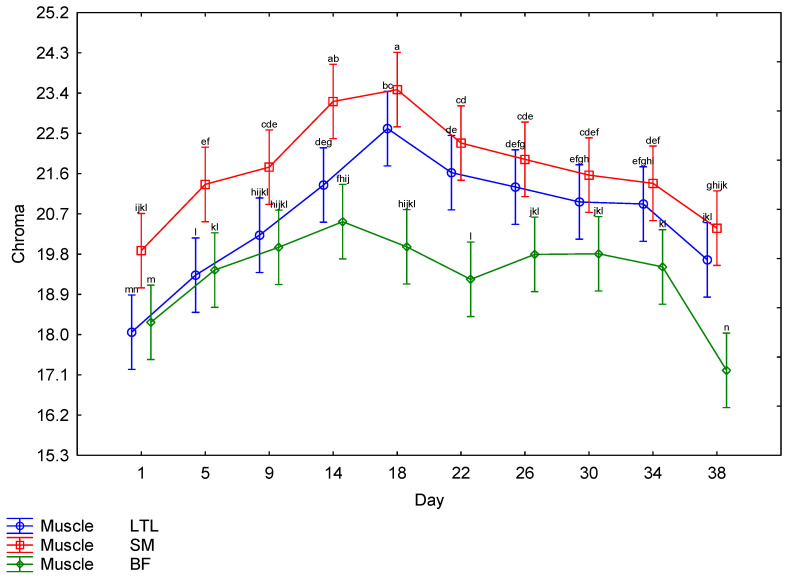
The effect of the interaction between muscle and days post-mortem on the chroma of giraffe meat during a 38-day ageing period. ^a–n^ Means with different superscripts differ from one another (*p* ≤ 0.05).

The interaction between muscle and days post mortem for the CIE L* values is illustrated in Figure 4. The BF showed higher L* values throughout the ageing period, with the lowest L* value for BF recorded on Day 1; there was a steady increase until Day 22, followed by a drop on Day 26, after which it maintained a similar lightness for the remainder of the ageing period. The LTL and SM both followed a similar trend, with the lowest L* values recorded on Day 1; there was a steady increase until Day 14, after which the readings remained relatively constant. There was a third-order interaction for the a* values. The males and females showed a very similar trend (Table 2); therefore, the effects of muscle and day (Figure 5) will be discussed further. The SM tended to have the highest a* values until Day 18, when the values started decreasing. The LTL showed trends similar to the SM but tended to have slightly lower values. The BF had the lowest a* values throughout the ageing period. The b* values were also affected by the third-order interaction. As both sexes followed a similar pattern (Table 2), only the muscle × day interaction will be discussed further (Figure 6). The LTL, SM and BF all increased steadily over the first 18 days of the ageing period, with the SM higher than the LTL and BF. All three muscles’ b* values declined over the rest of the ageing period. Although the third-order interaction for the hue-angle was significant (Table 2), the trends for the males and females were similar. Therefore, the second-order interaction (muscle × Day) is indicated in Figure 7. The BF had the highest hue-angles for both sexes throughout the ageing period, while the LTL and SM had similar values (Figure 7). All three muscles in both sexes had the lowest hue-angles on Day 1 of the ageing period and the highest values on Day 38, except for the BF in males, which had the highest value on Day 22. Although the chroma values showed a third-order interaction, as the trends were similar between the sexes (Table 2), the interaction between muscle and days is depicted in Figure 8. All muscles of both sexes tended to increase from Day 14 to Day 18 before declining again. The SM tended to have the highest values throughout most of the ageing period for both sexes. In contrast, the BF tended to have the lowest values for most of the ageing period.

## 4. Discussion

The effect of post mortem ageing on giraffe meat, irrespective of sex and muscle, has not yet been determined. Game culling is also often haphazard, with animals culled for management purposes rather than for optimal meat quality, and without accounting for the animal’s sex or other extrinsic and intrinsic factors that affect meat quality. Therefore, it may be valuable to focus on the broader impact of post mortem ageing on meat quality. It is well known that the pH of the meat during ageing influences not only its tenderness but also purge and cooking losses, as well as its colour [43,44,45].

The pH of the meat in this study decreased from Day 1 to Day 30. Although the total pH drop (~0.27 units) is relatively modest in absolute terms, it is meaningful in meat (Figure 1). A decrease in pH is typical of the changeover of muscle into meat caused by anaerobic glycolysis, and similar decreases have been observed in livestock [46,47,48] and several other game species, such as impala [14], eland [49], and springbok [50]. As ageing progresses, lactic acid bacteria produce lactic acid as they are favoured by the anaerobic conditions of wet-ageing meat [51]. Lactic acid typically forms on the surface of the meat; however, during structural breakdown during ageing, it may have penetrated deeper into the meat, causing the pH to decline further from Day 30. The lactic acid may result in a sour/off flavour, as found in springbok aged 28 days [52]. Towards the end of this study, a sour odour was noticed when the vacuum bags were opened. From a pH perspective, most biochemical ageing changes were complete by around Day 26. Extended ageing beyond that point did not cause a further pH change, though proteolytic tenderisation may continue independently of pH [53].

Although post mortem ageing is known to improve meat tenderness by promoting protease activity (calpains, cathepsins), which drives tenderisation [28,53], it also affects water-holding capacity—as pH moves further below the isoelectric point of myofibrillar proteins (~5.1–5.2), an increase in drip loss and cumulative purge loss from raw meat has been reported [28,54,55]. Purge loss causes bloody liquid to collect in the packaging, resulting in an unappealing packaged product. A high cumulative purge loss will also result in less moisture in the meat, less juicy cooked meat, and a loss of saleable weight. In addition, a significant amount of protein is also lost through purge loss [56]. The extent of purge loss is influenced by the meat’s pH and the proteolytic activities of the enzymes, which are themselves influenced by pH [28]. It affects the integrity of the cytoskeleton, decreasing the water-binding capacity [55].

The purge loss was affected only by the days post mortem and showed a negative relationship with pH: as pH decreased, water-holding capacity decreased, and more moisture was significantly expelled from the meat, with the largest increase in cumulative purge loss occurring from Day 1 to Day 5 (Figure 2). Early post mortem, as rigor mortis develops, the contraction of the myofibrillar proteins physically squeezes water from the muscle matrix [46,55,57]. This aligns with the period where the meat’s pH is stable at ~5.55 and near the isoelectric point of myosin (~5.1–5.4), where water-holding capacity is at its lowest relative to charge [55]. From Day 5, there was a more gradual increase in purge over the remainder of the ageing period. The curve flattens as the easiest-to-expel water has already been lost. Proteolytic degradation of the cytoskeleton during this period creates some new space for water within the myofibrillar structure, partially counteracting further loss [54,55,56]. During Days 26–38, there is a slow, continual rise in purge loss (~7.6% → ~9.0%): purge loss continues to increase but more slowly. Unlike pH (which plateaued by Day 26), purge loss continues to increase—on Day 38 it is still significantly higher than on Day 30. This suggests that structural degradation due to enzymatic action on the structural proteins (desmin, titin, nebulin) is responsible for the moisture loss in late ageing, as the ongoing proteolysis continues to release bound water even after pH has stabilised [54].

In contrast, in the literature [58], the purge loss percentage in chilled beef was found to plateau after the first 6–8 days, although this phenomenon was observed to take longer in different game species (14-day ageing period in impala [49]; 28-day ageing period in springbok [52]). The plateau in other species and the more gradual increase in giraffe may be due to the limited volume of liquid that can be released from within the cytoskeleton. In general, giraffe meat was found to have higher moisture loss than other game species during the physical analysis at 24 h post mortem across eight different muscles [39]. Over the ageing period of 38 days, the cumulative purge loss was ~9% for the giraffe’s meat. However, most other studies had shorter ageing periods, during which the purge typically plateaued after ±14 days. The cumulative purge loss of ≈7% at Day 14, despite being substantially higher than the 3.5% for blue wildebeest (*Connochaetes taurinus*) [59], was comparable with that of springbok (6.0% [50]) and the 6.5% for impala [49]. In summary, in this exploratory investigation on giraffe meat, by Day 38, nearly 9% of the product weight had been lost as purge—a significant economic and quality consideration. There is a clear trade-off between ageing for tenderness and losing saleable weight.

The cooking loss was not affected by the day of the ageing period; it was only affected by the muscle; the LTL had the lowest cooking loss, and the SM and BF both had higher cooking losses. Needham et al. [49] found that the cooking loss of impala meat was also unaffected by the ageing period. However, the losses were far lower than those recorded for giraffe. The cooking loss of the giraffe was comparable to that of the African Cape buffalo [15]. The LTL (38.0%) had a significantly lower cooking loss than the SM (41.1%) and BF (41.1%). Differences in cooking losses between the muscles may result from differences in muscle fibre type, reflecting their functional differences [60]; this would need to be confirmed by muscle fibre analysis.

The main aim of this study was to quantify the ageing period to optimum tenderness for three major giraffe muscles for both sexes. According to Miller et al. [61], consumers are willing to pay a premium price for meat of guaranteed tenderness, making tenderness arguably the most important qualitative characteristic of meat. Therefore, the classification of meat tenderness has been studied many times. Destefanis et al. [40] developed a three-category rating system after finding that a sensory panel could not distinguish between the five categories they initially tested. According to the three-category rating system, a WBSF value above 53 N is considered tough, and meat below 43 N is tender. Therefore, values between 43 N and 53 N are considered intermediate tender. The average Day 1 WBSF in the current study was below 43 N, which dropped progressively through the ageing period; thus, the muscles can be considered tender.

Although the WBSF was affected by a significant interaction between sex, muscle, and days post mortem, there were very few significant differences between the muscles for sex at any specific ageing point, all following a similar trend. The three muscles for both sexes all showed a decrease in WBSF from Day 1 to Day 22, after which they plateaued (Table 3). There was some fluctuation around this general trend. The *Longissimus thoracis et lumborum* (LTL)—The loin had an initial value of ~23.7 N, which dropped to ~18.0 N by Day 38, a ~24% reduction. The tenderisation was gradual and continuous. Most of the decrease had occurred by Days 14–18 (~20.5–20.7 N), with slight decreases thereafter.

The *Semimembranosus* (SM)—This hind leg muscle was initially the toughest at ~25.8 N and finishes at ~19.5 N, a ~24% reduction. However, the pattern was less consistent. There is a sharp initial drop from Day 1 (25.8 N) to Day 5 (24.9), and then a notable drop to Day 14 (~21.5 N), after which the values fluctuated—Day 18 increased to 22.7 N, then dropped to 20.3 N on Day 22. The high variability (large s.e. values) reflects the heterogeneous connective tissue content of this muscle, which proteolytic enzymes cannot break down (collagen is resistant to calpains/cathepsins) [46].

The *Biceps femoris* (BF)—Another hind leg muscle had an initial WBSF of ~25.4 N and ended at ~19.4 N (~24% reduction). This muscle tenderised relatively quickly to ~19.5 N by Day 14, and then barely changed (non-significant) through to Day 38. This suggests that the BF reached its ageing potential early, likely because its fibre structure and enzyme profile allow rapid proteolysis.

When evaluating the meat in general, it had an average Day 1 WBSF of 25.0 ± 0.73 N and a Day 38 value of 18.9 ± 0.73 N, which did not differ from the average Day 22 value (19.0 ± 0.73 N). Although the meat was considered tender (<43 N) from Day 1, ageing progressively improved the tenderness until Day 22. Commercially, the LTL of cattle is typically aged for at least 14 days for improved tenderness, and some butchers choose to age it for 35 days or longer. In game species, the optimum tenderness was attained by eight days post mortem for springbok (*Antidorcas marsupialis*) [52], after eight days for impala (*Aepyceros melampus*) [49], and after 25 days in buffalo (*Syncerus caffer caffer*) [15], whilst eland (*Taurotragus oryx*) was still tough after 35 days of ageing [14].

Surface colour is an important factor in consumer preferences when purchasing meat, as it indicates freshness [62,63,64]. As the surface colour was significantly affected by the ageing period, it is important to consider this effect when ageing meat, as discolouration may negate the positive effect of improved tenderness on the price a consumer is willing to pay [65,66,67]. However, this would only be applicable to bloomed meat. The colour stability is affected by species and muscle [68]. Therefore, it is necessary to investigate each species when investigating the ideal ageing period. The colour parameters were all affected by interactions. The L* values were only affected by a second-order interaction between muscle and days post mortem. The other colour parameters were all affected by a third-order interaction between sex, muscle and days post mortem. The L* values increased from Day 1 to Day 22 for the LTL and to Day 14 for the SM and BF, indicating a general increase in lightness across all muscles (Figure 4). While an increase in lightness is usually seen as an improvement in game meat, as it is generally darker than beef [22], giraffe meat was much lighter than other game species on Day 1 [49]. Despite most publications reporting a consumer preference for lighter meat [69,70,71], the preference is for bright red over pale red [72,73,74], which may mean that consumers will begin to discriminate against meat with an L* value above a certain point, as may be the case with the BF meat, although no specific cut off could be found in the literature. Therefore, a further increase in lightness may not be desirable to the consumer. The a* values of all muscles from both sexes increased slightly until 18 days, after which they decreased significantly, indicating a decline in meat redness. The b* values increased until Day 18 in males and Day 14 in females, after which they remained high, indicating an increase in the yellowness of the meat at the beginning of the ageing period, which is undesirable. This resulted in a gradual increase in the hue-angle throughout the ageing period, indicative of unacceptable discolouration [22]. The chroma also peaked on Day 18 before declining, indicating that the meat had the highest saturation on that day. Overall, this showed that post mortem ageing positively affected the surface colour of giraffe meat, increasing saturation up to Day 18. However, this coincided with an increase in yellow and, consequently, in hue-angle. However, as consumer preference is for brighter, redder meat [65,75], this still constitutes an improvement. If aged for more than 18 days, the meat will become duller and browner in colour when bloomed, and by the end of the ageing period, excessive discolouration was observed in some steaks, which may be due to microbial spoilage [22].

## 5. Conclusions

This exploratory study aimed to determine the ideal ageing period to optimise tenderness for the LTL, SM and BF muscles of both male and female giraffe. A major limitation of this study is that the animals were sourced from a single farm with similarly aged giraffes (a small genetic pool and a singular environment), and it is suggested that similar studies be conducted on a larger sample of more diverse animals. The most important finding is that the optimal ageing period differs by muscle. The BF achieves near-maximum tenderness by Day 14, while the LTL and SM continue improving (albeit slowly) through to Days 26–30. This has direct practical implications—if you are ageing a whole carcass uniformly, some muscles will be “done” long before others.

While tenderness improved post mortem, the percentage moisture loss progressively increased throughout the ageing period, and the surface colour of the muscle (after blooming) improved until Day 18 but was negatively affected by further ageing. pH declines gradually over 38 days (primarily Days 9–26), reducing water-holding capacity and driving increasing purge losses throughout the entire period. Meanwhile, the falling pH optimises conditions for endogenous proteases (particularly cathepsins, which are most active at lower pH), driving the tenderisation seen in the WBSF data. Practically, the ideal ageing time seems to be around Days 14–22—by then, most of the tenderisation benefit across all muscles has occurred, and pH has dropped meaningfully, but purge losses are still moderate (7–7.5%) rather than the ~9% seen at Day 38.

However, as ageing is known to affect the sensory quality and microbial safety of meat, it is recommended that the effects be investigated on the sensory (trained panel and/or consumer) profile (perceived juiciness, flavour and overall acceptability) and the microbial fauna of wet-aged giraffe meat. Additional analyses could also include the effect of wet-ageing on lipid and protein oxidation.

## Figures and Tables

**Table 1:** Experimental layout of the post mortem ageing trial per main effect (sex and ageing period).

Number of Animals	Sex	Ageing Period (Days Post Mortem)									
8	Male	1	5	9	14	18	22	26	30	34	38
7	Female	1	5	9	14	18	22	26	30	34	38

**Table 2:** Level of statistical significance (*p*-values) for the main effects of sex, muscle and day (post mortem) and their interaction with the physical parameters of giraffe meat during the 38-day ageing period.

Parameter	Age	Sex	Muscle	Day	S × M	S × D	M × D	S × M × D
pH	0.260	0.155	0.669	<0.001	0.250	0.401	0.146	0.083
Purge loss (%)	0.242	0.968	0.254	<0.001	0.826	0.563	0.490	0.488
Cooking loss (%)	0.078	0.260	<0.001	0.318	0.502	0.658	0.686	0.208
Shear force (N)	0.148	0.177	0.051	<0.001	0.300	0.081	0.008	0.002
L*	0.080	0.727	<0.001	<0.001	0.148	0.203	<0.001	0.533
a*	0.134	0.868	<0.001	<0.001	0.655	0.602	<0.001	<0.001
b*	0.300	0.732	0.001	<0.001	0.755	0.095	<0.001	0.030
Hue-angle	0.082	0.805	<0.001	<0.001	0.462	0.003	<0.001	<0.001
Chroma	0.278	0.960	<0.001	<0.001	0.560	0.895	<0.001	<0.001

**Table 3:** Mean WBSF (N) indicating the interaction between muscle and day of ageing.

Day	LTL	SM	BF
1	23.7 ^bcd^ ± 6.22	25.8 ^a^ ± 6.52	25.4 ^ab^ ± 5.70
5	22.7 ^defgh^ ± 6.94	24.9 ^abc^ ± 7.10	20.1 ^klmno^ ± 6.05
9	22.1 ^defghijk^ ± 6.92	23.7 ^bcd^ ± 5.85	23.1 ^cdefg^ ± 6.62
14	20.5 ^iklm^ ± 5.30	21.5 ^efghijkl^ ± 5.79	19.5 ^lmno^ ± 4.51
18	20.7 ^klm^ ± 7.49	22.7 ^defhi^ ± 5.69	20.4 ^jklmno^ ± 6.43
22	18.4 ^no^ ± 5.78	20.3 ^jklmno^ ± 4.85	18.5 ^mno^ ± 3.49
26	18.9 ^mno^ ± 5.27	20.6 ^gklmn^ ± 5.15	19.4 ^lmno^ ± 4.33
30	18.1 ^o^ ± 4.88	20.6 ^fghijklmn^ ± 4.22	19.6 ^klmno^ ± 4.90
34	20.7 ^hijk;mno^ ± 6.55	21.5 ^efghijkl^ ± 5.63	20.5 ^hijklmno^ ± 4.65
38	18.0 ^o^ ± 4.97	19.5 ^klmno^ ± 4.13	19.4 ^lmno^ ± 9.03

^a–o^ Means (±s.e.) with different superscripts differ from one another (*p* ≤ 0.05).

## Data Availability

The data presented in this study are available on request from the corresponding author due to (privacy reasons).

## Supplementary Materials

The following supporting information can be downloaded at https://www.mdpi.com/article/10.3390/foods15071236/s1: Table S1: Data showing the minimal impact of sex on the WBSF, CIE *a**, CIE *b**, hue-angle, and chroma measurements.

## Abbreviations

The following abbreviations are used in this manuscript:

LTL	*Longissimus thoracis et lumborum*
SM	*Semimembranosus*
BF	*Biceps femoris*
WBSF	Warner–Bratzler shear force
N	Newton
