# Supplementary material for: Wet Ageing of Chilled Young-Giraffe (Giraffa camelopardalis angolensis) Meat as Influenced by Sex and Muscle
Source: Foods. 2026 Apr 4;15(7):1236. doi: 10.3390/foods15071236 (PMC13073653; doi:10.3390/foods15071236)
Supplement: Supplementary file 1 [file foods-15-01236-s001.zip › foods-4209803-supplementary.pdf]

Data showing the minimal impact of sex on the WBSF, CIE *a**, CIE *b**, hue-angle, and chroma measurements

WBSF:

Random effects: (1|Nr)+(1|Nr:Muscle)+(1|Nr:Day)
Degrees of freedom: Kenward-Rogers

	1<br>Sum Sq	2<br>Mean Sq	3<br>Num DF	4<br>Den DF	5<br>F value	6<br>**p value**
**age**	57.39	57.39	1	12	2.39	0.148
sex	49.34	49.34	1	12	2.06	0.177
Muscle	159.85	79.92	2	26	3.33	0.051
Day	2477.19	275.24	9	117	11.48	0.000
sex*Muscle	60.55	30.28	2	26	1.26	0.300
sex*Day	382.63	42.51	9	117	1.77	0.081
Muscle*Day	1055.35	58.63	18	2484	2.44	0.001
sex*Muscle*Day	968.31	53.80	18	2484	2.24	0.002

a*:

Random effects: (1|Nr)+(1|Nr:Muscle)+(1|Nr:Day)
Degrees of freedom: Kenward-Rogers

	1<br>Sum Sq	2<br>Mean Sq	3<br>Num DF	4<br>Den DF	5<br>F value	6<br>**p value**
age	6.44	6.44	1	12	2.59	0.134
**sex**	0.07	0.07	1	12	0.03	0.868
Muscle	382.26	191.13	2	26	76.85	0.000
Day	235.48	26.16	9	117	10.52	0.000
sex*Muscle	2.14	1.07	2	26	0.43	0.655
sex*Day	18.27	2.03	9	117	0.82	0.602
Muscle*Day	449.53	24.97	18	2031	10.04	0.000
sex*Muscle*Day	155.28	8.63	18	2031	3.47	0.000

b*:

Random effects: (1|Nr)+(1|Nr:Muscle)+(1|Nr:Day)
Degrees of freedom: Kenward-Rogers

	1<br>Sum Sq	2<br>Mean Sq	3<br>Num DF	4<br>Den DF	5<br>F value	6<br>**p value**
age	1.76	1.76	1	12	1.17	0.300
**sex**	0.19	0.19	1	12	0.12	0.732
Muscle	27.13	13.57	2	26	9.02	0.001
Day	915.91	101.77	9	117	67.65	0.000
sex*Muscle	0.85	0.43	2	26	0.28	0.755
sex*Day	23.12	2.57	9	117	1.71	0.095
Muscle*Day	117.80	6.54	18	2031	4.35	0.000
sex*Muscle*Day	46.48	2.58	18	2031	1.72	0.030

Hue:

	Random effects: (1|Nr)+(1|Nr:Muscle)+(1|Nr:Day)<br>Degrees of freedom: Kenward-Rogers					
	1<br>Sum Sq	2<br>Mean Sq	3<br>Num DF	4<br>Den DF	5<br>F value	**6**<br>**p value**
age	58.47	58.47	1	12	3.60	0.082
**sex**	1.03	1.03	1	12	0.06	0.805
Muscle	4882.69	2441.35	2	26	150.14	0.000
Day	7363.05	818.12	9	117	50.31	0.000
sex*Muscle	25.85	12.93	2	26	0.79	0.462
sex*Day	446.43	49.60	9	117	3.05	0.003
Muscle*Day	965.56	53.64	18	2032	3.30	0.000
sex*Muscle*Day	1000.88	55.60	18	2032	3.42	0.000

Chroma:

	Random effects: (1|Nr)+(1|Nr:Muscle)+(1|Nr:Day)<br>Degrees of freedom: Kenward-Rogers					
	1<br>Sum Sq	2<br>Mean Sq	3<br>Num DF	4<br>Den DF	5<br>F value	**6**<br>**p value**
age	3.16	3.16	1	12	1.29	0.278
sex	0.01	0.01	1	12	0.00	0.960
Muscle	187.89	93.94	2	26	38.42	0.000
Day	273.13	30.35	9	117	12.41	0.000
sex*Muscle	2.90	1.45	2	26	0.59	0.560
sex*Day	10.24	1.14	9	117	0.47	0.895
Muscle*Day	441.23	24.51	18	2034	10.03	0.000
**sex*Muscle*Day**	246.58	13.70	18	2034	5.60	0.000
